# Vitamin D: Mechanism of Action and Biological Effects in Uterine Fibroids

**DOI:** 10.3390/nu13020597

**Published:** 2021-02-11

**Authors:** Daniele Vergara, William H. Catherino, Giuseppe Trojano, Andrea Tinelli

**Affiliations:** 1 Department of Biological and Environmental Sciences and Technologies (DiSTeBA), University of Salento, strada Prov. le Lecce-Monteroni, 73100 Lecce, Italy; 2 Department of Obstetrics and Gynecology, Uniformed Services University of the Health Sciences, Bethesda, MD 20814, USA; william.catherino@usuhs.edu; 3 Program in Reproductive Endocrinology and Gynecology, Eunice Kennedy Shriver National Institute of Child Health and Human Development, National Institutes of Health, Bethesda, MD 20814, USA; 4 Department of Obstetrics and Gynecology, “Madonna delle Grazie” Hospital, 75100 Matera, Italy; giutrojano@gmail.com; 5 Department of Obstetrics and Gynecology, “Veris delli Ponti” Hospital, Scorrano, 73020 Lecce, Italy; andreatinelli@gmail.com; 6 Division of Experimental Endoscopic Surgery, Imaging, Technology and Minimally Invasive Therapy, Vito Fazzi Hospital, 73100 Lecce, Italy; 7 Laboratory of Human Physiology, Phystech BioMed School, Faculty of Biological & Medical Physics, Moscow Institute of Physics and Technology (State University), Dolgoprudny, 141701 Moscow, Russia

**Keywords:** uterine fibroids, leiomyoma, vitamin D, vitamin D receptor (VDR), cell signaling

## Abstract

Uterine fibroids (UFs) are the most common benign gynecological tumors. It was estimated that fifty percent of women presenting with UFs has symptomatology that negatively influences their quality of life. Pharmacological and/or surgical treatments are frequently required, depending on the woman’s desire to preserve fertility, with a high impact on healthcare costs. Generally, the use of currently available pharmacological treatments may lead to side effects. Therefore, there is a growing interest in a natural and safe approach for UFs. In recent years, epidemiological studies reported a vitamin D deficiency in patients with UFs raised interest in the potential biological effects of vitamin D supplementation. In vitro studies proved vitamin D efficacy in inhibiting UFs growth by targeting pathways involved in the regulation of various biological processes, including proliferation, extracellular matrix (ECM) remodeling, DNA repair, signaling and apoptosis. However, clinical studies supported only in part the beneficial effects of vitamin D supplementation in reducing UFs growth and tumor volume. Randomized controlled trials and large population studies are mandatory as the potential clinical benefits are likely to be substantial.

## 1. Introduction

Uterine fibroids (UFs), also known as uterine leiomyomas or myomas, are the most frequent benign tumors in women of reproductive age. More precisely, UFs are monoclonal tumors derived from smooth muscle tissue, the myometrium [1]. UFs incidence ranges between 30 and 70% in women of reproductive age, increases at the end of the reproductive age and is higher in some ethnic groups, especially the Afro-Caribbean. Furthermore, UFs can undergo growth inhibition or spontaneous regression after menopause [1]. Histologically, UFs structure is composed of a fibrovascular pseudocapsule surrounding a tissue consisting of smooth muscle cells, fibroblasts, and connective tissue [2,3]. Although about 50% of cases are asymptomatic, the quality of life (QoL) of women presenting with UFs may often be impaired by symptoms that include menometrorrhagia, anemia, bladder pressure (pollakiuria and/or urinary retention) or rectal pressure (constipation), as well as a sense of weight and dyspareunia [4]. Symptoms are closely related to the number, location and size of the tumor. Furthermore, increasing evidence shows that UFs can negatively affect fertility [1,4].

The last decade has seen many important advances in understanding the molecular background of these tumors, including the identification of specific driver mutations by high-throughput studies [5]. Current efforts are now aimed at identifying and characterizing their specific biological and clinical value [6]. In addition to this genomic background, UFs are characterized by an intense production of extracellular matrix (ECM) with high levels of collagen and fibronectin produced by local fibroblasts [1]. This is a distinctive feature of this tumor type, and current efforts are now aimed at better defining the compositional differences in the ECM in myometrium and UFs [7,8]. In light of its critical role, ECM represents a standalone target in UFs therapy [9]. Overall, genomic mutations and alterations of the ECM microenvironment should be considered as a combined driving force of UFs growth.

In designing the proper treatment for symptomatic UFs, clinicians should take into account their location, number and size, the patient’s comorbidities and their desire to maintain fertility [10]. The first approach is the use of medical treatments, including hormonal contraceptives, gonadotropin-releasing hormone analog (GnRHa), and selective progesterone receptor modulators (SPRM) [1,11,12,13,14,15,16,17].

Surgical approaches include myomectomy, hysterectomy, magnetic resonance-guided focused ultrasound surgery, and uterine artery embolization [18,19,20,21]. Hysterectomy remains the only definitive treatment, while the other surgical interventions show a 3% to 32% reintervention rate within 5 years, depending on the technique [22,23,24].

Considering the side effects of both medical and surgical treatments and the reintervention rate, a safer and cost-effective approach is highly desirable. This review focuses on the biological effects of vitamin D in UFs and on the molecular mechanisms underlying vitamin D action. We also discuss some of the results that emerged from the combination of vitamin D with other compounds.

## 2. Synthesis and Metabolism of Vitamin D

Vitamin D is the common name used to indicate two lipophilic steroidal compounds: ergocalciferol (vitamin D2) and cholecalciferol (vitamin D3) [25]. These two molecules differ structurally for a double bond and a methyl group present only in the vitamin D2 [26] and for their bioavailability, which is higher for the vitamin D3 form. The majority of vitamin D3 (80–90%) is produced by the skin after exposure to ultraviolet (UVB) radiation between 280 and 315 nm, and only a small amount comes from dietary intake [27]. Vitamin D2 mainly comes from vegetables, fungi and yeasts, while vitamin D3 comes almost exclusively from animal products such as fish, meat, milk, and eggs [28]. After skin synthesis and/or intestinal absorption, both vitamin D2 and D3 undergo the same enzymatic conversions, first in the liver and then in the kidney. In the liver, vitamin D is converted to 25-hydroxyvitamin D (25(OH)D or calcidiol) by 25-hydroxylase and this conversion is poorly regulated and almost exclusively dependent on vitamin D levels [26]. To become active, 25(OH)D must be converted to 1,25-dihydroxyvitamin D (1,25(OH)_2_D or calcitriol). This transformation occurs mainly in the kidney but also in all tissues where the enzyme 25-hydroxyvitamin D_3_-1α-hydroxylase (encoded by CYP27B1) is expressed. The concentration of calcium and phosphate regulates the synthesis of this enzyme through negative feedback, which is also mediated by the parathyroid hormone (PTH) levels [29]. 1,25(OH)_2_D limits its own activity by inducing the expression of a mitochondrial inner membrane cytochrome P450 enzyme (encoded by CYP24A1 gene).

The action of calcitriol is mediated by its interaction with the vitamin D receptor (VDR), a member of the superfamily of nuclear receptors. VDR is present in almost all human tissues and consequently involved in the regulation of several biological functions [30]. Upon interacting with 1,25(OH)_2_D, VDR dimerizes with the retinoic acid receptor (RXR) and binds to vitamin D response elements (VDRE) in the promoter sequences of genes that are induced or repressed by VDR. Gene expression is also dependent on the interaction with coactivators and corepressors to modulate multiple gene regulatory networks in a cell-type-specific manner [31].

Physiologically, vitamin D effects are mainly associated with the regulation of calcium and phosphorus homeostasis in the serum as well as in the intestine, bone, parathyroid and kidney [32]. However, the ubiquitous expression of the VDR in several human tissues significantly extends the effect of vitamin D to the regulation of hormone production, cell proliferation, differentiation as well as modulation of the immune system [28,32]. Because of these functions, vitamin D deficiency is associated with various pathological conditions, including cardiovascular disease, immune disorders, and several types of cancer [33,34,35]. In this scenario, many studies have reported the association between vitamin D status and UFs [36,37,38].

## 3. Correlation between Vitamin D and Fibroids

It is well recognized the role of diet and micronutrients in the biology and pathophysiology of UFs [39]. In detail, several studies have demonstrated a close correlation between vitamin D deficiency and the potential development of UFs in the Caucasian, African American, Indian and Chinese populations [40,41,42,43,44].

In 2013, the analysis of Baird and colleagues on 25(OH)D serum levels and UFs estimated 32% reduced odds of fibroids in women with physiological vitamin D levels, compared with those with vitamin D insufficiency (below 20 ng/mL) [40]. In this work, fibroid status was determined by ultrasound screening of premenopausal women, 620 blacks and 416 whites. Conversely, data from Mitro and colleagues suggested that insufficient serum 25(OH)D was significantly associated with the odds of UFs in white, but not black, women [41].

In the study by Paffoni and colleagues, 128 Italian women with UFs were compared with 256 healthy controls [42]. Women were grouped into 3 different groups: vitamin D deficient (<10 ng/mL), insufficient (10–19.9 ng/mL), and sufficient (≥20 ng/mL). These values were determined according to the World Health Organization recommendations but are different from the cut-point that was set at 30 ng per mL by the Endocrine Society committee [45]. By considering World Health Organization recommendations, women with UFs showed significantly lower 25(OH)D levels than the healthy subjects (18.0 ± 7.7 ng/mL vs. 20.8 ± 11.1 ng/mL; *p* = 0.01) [42]. In the case–control study of Li and collaborators, a high prevalence of hypovitaminosis D was observed among women of reproductive age in China. Moreover, lower serum 25(OH)D levels were found in UFs patients compared to females without fibroids [43].

A more recent observational study, including eastern Indian individuals [44], confirmed only in part the results of Paffoni and colleagues. In this work, lower serum levels of 25(OH)D were observed in women with UFs (*n* = 72) compared to controls (*n* = 72). However, data from this study did not reveal any significant correlation between the fibroid number and size with serum 25(OH)D level suggesting that vitamin D levels are not probably associated with the extent of the disease.

In a recent systemic review and meta-analysis, Mohammadi and colleagues analyzed pooled data from none studies with a total of 1730 participants (835 patients with UFs and 895 controls). Summarizing the data, the authors concluded that lower vitamin D levels are correlated with a higher probability of UFs diagnosis [46]. However, they also suggested that larger studies are necessary to obtain definitive results and to address some study design limitations, i.e., small sample size and ethnicity. For instance, several studies report that the frequency of vitamin D deficiency is approximately 10 times higher in African-Americans than in Caucasians [47]. Factors affecting epidermal vitamin D synthesis involve either the time of exposure to sunlight or the skin complexion. As the skin pigmentation may reduce the absorption of UVB in the black population [48], the elevated frequency of UFs could be correlated with the higher incidence of vitamin D deficiency. Other molecular determinants of vitamin D status should also be considered. Recent studies have also demonstrated a correlation between the occurrence of UFs and genetic polymorphisms involved in vitamin D metabolism [49]. In particular, in a large cohort of African American women, UF risk was positively associated with two single nucleotide polymorphisms (SNPs) rs1280043 near the gene 7-dehydrocholesterol reductase (DHCR7) and rs6058017 in Agouti-signaling protein (ASIP) [50].

## 4. Mechanisms Underlying the Action of Vitamin D in Uterine Fibroids

**Figure 1:**
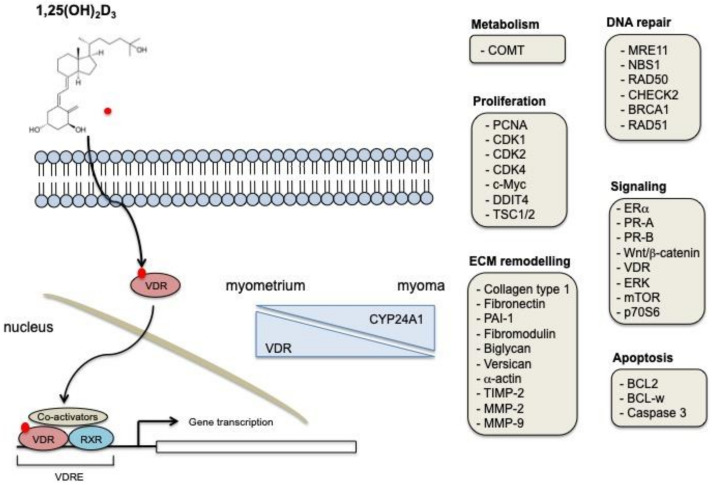
Biological mechanisms of vitamin D action in UFs. 1,25-dihydroxyvitamin D is the biologically active form of vitamin D. It exerts its cellular effects through the binding to VDR. The VDR-RXR complex binds to specific DNA sequences, thus regulating the expression of specific target genes. Vitamin D receptor (VDR) expression decreases in UFs samples, while CYP24A1 expression is augmented. Some of the genes and cellular processes modulated in UFs after vitamin D treatment are shown.

There is increasing evidence that vitamin D physiological action is driven by the activation of multiple cellular pathways, including proliferation, apoptosis, DNA repair, ECM deposition, and signaling. Specifically, its action in UFs cells is strictly correlated to the ability of vitamin D to control the expression of VDR. Moreover, dysregulation of vitamin D metabolic enzymes CYP27B1 and CYP24A1 that was observed in UFs, compared to normal myometrium, could locally regulate vitamin D action [51] (Figure 1).

To understand the biological role of this molecule in the growth of fibroids, Blauer and colleagues performed a study showing that 1,25(OH)_2_D_3_ inhibits the growth of both leiomyoma cells and myometrial cells derived from the tissue of premenopausal women undergoing hysterectomy. In this study, calcitriol showed dose-dependent growth inhibition of UFs, on both myoma and leiomyoma cells [52]. This was one of the first studies to demonstrate the effect of vitamin D on leiomyoma cells, but with some limitations. In fact, evidence of a specific effect on leiomyoma cells lacks as an antiproliferative action of vitamin D was also observed in myoma cells even at the physiological concentration of D. Moreover, the authors used a simple colorimetric method to detect cell viability and did not characterize the biological and molecular causes of reduced crystal violet staining.

This preliminary result stimulated research on the potential antiproliferative effects of vitamin D on leiomyoma cells. In the work of Sharan and collaborators, the biological effects of calcitriol were evaluated in immortalized human uterine fibroid cells (HuLM) cells in a concentration and time-dependent manner. Vitamin D treatment reduced the growth of HuLM, and this was associated with a reduced expression of cell proliferation nuclear antigen (PCNA), beta-cell lymphoma 2 (Bcl-2), Bcl-w, cyclin-dependent kinase 1 (CDK1) and catechol-O-methyltransferase (COMT) [53].

More recently, the effects of vitamin D on pathways that drive cell proliferation have been described. Experimental studies led to the suggestion that modulation of estrogen signaling might be involved in explaining vitamin D effects on proliferation. UFs are hormonally regulated, and one important factor in the development and growth of fibroma is the over-expression of receptors for progesterone (PR) and estrogens (ER), compared to normal myometrium [54]. In this regard, vitamin D3 showed inhibitory capacities on the expression of ER and PR in a dose-dependent manner in leiomyoma cells [55]. In HuLM cells, vitamin D has the potential to reduce nuclear ERα, ΠΡ–A, ανδ levels as well as the expression of steroid receptor coactivator (SRC)1, (SRC)2, and (SRC)3. This result emphasizes the antiestrogenic/antiprogesteronic role of VDR. Moreover, 1,25(OH)_2_D_3_ also has an effect on VDR expression, whereas the expression levels of nuclear RXR-α were unaffected by treatment [56]. Results from normal tissues or other tumor types may provide a generalizable insight into the mechanisms that regulate vitamin D effects against ER. In breast cancer, vitamin D regulates the proliferation of luminal-like models (ER+) and reduces the ERα expression [56]. In normal myometrial cells, vitamin D modulates the expression of the inflammatory markers and ERα when cells are co-cultured with human monocyte lineage (THP1) cells [57]. Summarizing these data, we can conclude that vitamin D has an effect on the regulation of PR and ERα in different tissues and given the central role of both nuclear receptors in the control of cell proliferation and other biological processes, these results are quite remarkable. As estrogens can signal through nuclear receptors and a G-protein–coupled estrogen receptor, further studies are needed to define the regulation of vitamin D on estrogen signaling. Moreover, we still lack knowledge of vitamin D effects on estrogen synthesis through aromatase in UFs (i.e., reduction of aromatase expression).

In addition to targeting ER signaling, the effects of vitamin D on the wingless-type (Wnt)/β-catenin pathway were also supported by experimental studies [58]. Among the various molecular effects of this pathway with relevance to UFs physiology, Wnt ligands WNT11 and WNT16B act as paracrine signals secreted from hormonal positive UFs cells to activate β-catenin signaling in leiomyoma stem cells. This induces the activation of a β-catenin gene program supporting cell proliferation and tumorigenesis [59]. In vitro, calcitriol reduced in a concentration-dependent manner the expression of β-catenin as well as the expression of the Wnt signaling associated proteins such as Wnt4, Wnt1-inducible-signaling pathway protein 1 (Wisp1), and FEN1. Moreover, calcitriol treatment suppressed the Wnt/β-catenin downstream signaling pathways mTOR. VDR-knockdown cells with reduced levels of VDR showed higher levels of phosphorylated mTOR and increased ECM protein expression [60]. A PCR-array study in human uterine leiomyoma primary cells identified a large set of Wnt-related genes modulated after calcitriol treatment. In detail, VDR activation suppresses the activation of Wnt-related genes involved in the regulation of biological processes, including tissue polarity, cell migration, cell cycle, cell growth, and proliferation [60]. Therefore, researchers conclude that by inhibiting WNT–β-catenin signaling, vitamin D may have a therapeutic benefit in UFs.

More recently, Corachán and collaborators evaluated the effects of vitamin D on the Wnt/β-catenin and TGFβ signaling pathways in human uterine leiomyoma primary (HULP) cells isolated from mediator complex subunit 12 (MED12) -mutated and wild type leiomyomas. Expression of Wnt/β-catenin and TGF β pathway genes is, in fact, significantly increased in MED12-mutated leiomyomas. The authors of this study demonstrated that vitamin D exerted its antiproliferative action irrespective of MED12 mutation status [61].

Several other beneficial effects of vitamin D have been described, including a potential role in the modulation of VDR and ECM components. In this study, the authors demonstrated that UFs expressed lower levels of VDR than adjacent normal myometrium. In vitro, this downregulation is reverted by a dose-dependent calcitriol exposure of HuLM. Overall, this means that vitamin D causes upregulation of VDR in cultured leiomyoma cells but does not explain the molecular mechanisms for regulation of VDR expression (i.e., transcriptional or post-transcriptional) and does not clarify how this influence the response to vitamin D (i.e., reduced proliferation, increased expression of vitamin D target genes). In the same work, treatment with calcitriol was also associated with a significantly reduced protein expression of ECM-associated collagen type 1, fibronectin, and plasminogen activator inhibitor-1 (PAI-1), as well as reduced mRNA and protein expressions of proteoglycans such as fibromodulin, biglycan, and versican in HuLM cells. Calcitriol also demonstrated its biological efficacy by reducing the excessive synthesis and deposition of disorganized structural smooth muscle actin fibers [62].

Calcitriol effects on ECM remodeling proteins might also be mediated through the mRNA, and protein downregulation of matrix metalloproteinases (MMPs)-2 and MMP-9 expression, and a concomitant increased expression of tissue inhibitors of matrix metalloproteinase (TIMP)-2 [63].

It was recently demonstrated that calcitriol has a direct effect on the regulation of DNA repair proteins. In UFs, DNA repair mechanisms can be dysfunctional and associated with a downregulation of DNA repair protein members belonging to DNA double-strand breaks (DSBs) (MRE11, NBS1, RAD50), mediators and effectors (CHECK2, BRCA1, RAD51) compared to the myometrium. In myometrial cells, the knock-down of VDR by short hairpin RNA (shRNA) induced DNA double-strand breaks, accumulation and DNA damage response (DDR) defects, thus suggesting a role for the calcitriol/VDR axis in the development of UFs via the DNA repair pathway. Administration of calcitriol in HuLM cells significantly decreased DNA damage and restored DDR concomitant with VDR induction. This can be ascribable to an increased rate of mRNA synthesis, as demonstrated by the significant induction of VDR transcript [64]. In addition, a study by Elkafas and collaborators showed that calcitriol treatment attenuated the DNA damage load in myometrial stem cells (MMSCs) exposed to the endocrine-disrupting chemical diethylstilbestrol (DES) [65].

## 5. Vitamin D in the Treatment of Fibroids

Preclinical and clinical studies investigated the response of normal myometrium and uterine fibroids to vitamin D treatment. In mice, 25(OH)D deficiency was associated with a pre-fibroid status in the myometrium. This was characterized by an increased expression of sex steroid receptors, increased expression of proliferation-related genes, increased DNA damage, and the promotion of fibrosis. 25(OH)D deficiency also enhanced inflammation and promoted immunosuppression through regulatory T cells (Tregs) expansion in murine myometrium compared to a control group [66].

A preclinical study described the effect of oral vitamin D supplementation in Eker rats carrying a germline mutation in one allele of the Tsc-2 tumor-suppressor gene. This animal model and derived cell lines share some phenotypic and biochemical characteristics with human UFs, including estrogen and progesterone receptor expression and responsiveness to steroid hormones. The authors observed that treatment with vitamin D was followed by tumor growth and cell proliferation inhibition through downregulation of PCNA, cyclin D1, c-Myc, CDK1, CDK2 and CDK4 protein expression. Treated mice also showed a reduction in tumor size. This could be related to the apoptosis induction, confirmed by the expression reduction of Bcl-2, Bcl-xl, ERα and both PR-A and PR-B after vitamin D treatment [67]. Other experimental studies support this effect of vitamin D in vivo. The effect of calcitriol and its analog paracalcitriol, an analog of vitamin D with lower calcemic activity, was also studied and compared. Both molecules showed efficacy in reducing UFs volume in nude mice implanted with Eker rat tumor-derived ELT-3 cells, compared to untreated controls [68]. In a recent study, Corachán and collaborators investigated the effects of short- and long-term vitamin D treatment on UFs in vivo. Human leiomyomas were collected from patients and implanted in ovariectomized NOD-SCID mice and treated with vitamin D (0.5 μg/kg/d or 1 μg/kg/d) or vehicle for 21 or 60 days. Long-term treatment with vitamin D showed an antiproliferative, antifibrotic, and proapoptotic effect [69].

In reason of the interest in vitamin D as a potential UFs treatment, several clinical studies were performed in the last years. An open-label clinical trial on vitamin D was carried out by Ciavattini and colleagues on 108 women with “small burden” UFs and vitamin D deficiency (<30 ng/mL) [70]. Patients were divided into two groups: a group (*n* = 53) treated with vitamin D (50,000 IU per week for 8 weeks and then 2000 IU per day for 12 months) and an untreated control group (*n* = 55). At the end of the study, the treated group showed UFs growth inhibition and a lower rate of surgical or medical treatment compared to the control group. On the other hand, the control group showed UFs growth with an increased rate of need for surgical treatment due to the severity of the symptoms [70].

In a prospective, double-blind study, 69 women with UFs and vitamin D deficiency were divided into 2 groups (treated and placebo). The first group received an oral administration of 50,000 IU of vitamin D every 2 weeks for a total of 10 weeks. Six months after the intervention, in addition to the significant increase in vitamin D levels, the treated group showed a significant reduction in UFs volume compared to the control group [71].

In a recently published randomized clinical trial (RCT), women with UFs and vitamin D deficiency (level less than 30 ng/mL) received 50,000 IU oral vitamin D or a placebo for 12 weeks. Although the UFs volume did not decrease after treatment, the authors observed that vitamin D consumption induced a block of UFs tumor growth in the experimental group [72].

In 2019, the first oral vitamin D supplementation in combination with ulipristal acetate (UPA) was studied in 2 women (37 and 49 years old, respectively) with symptomatic UFs. Both patients were treated daily for 3 months with 7000 IU vitamin D and 5 mg UPA. In the first woman, the combined treatment showed a significant reduction in symptoms (pain, pressure, and urinary frequency) in addition to a 47.8% reduction in UFs volume. In the second woman, most of the symptoms disappeared, and there was a reduction in UFs volume of 63.3% [73]. An in vitro study tried to investigate the biological rationale at the basis of this combination. The authors demonstrated that the combined treatment of HuLM cells with UPA and calcitriol enhanced the antiproliferative effects of UPA alone [74]. Several biological processes, including apoptosis, ECM remodeling, and inflammation, were significantly modulated by the combination. However, as the authors did not investigate the effects of vitamin D alone, concerns remain about the biological efficacy of this combination.

In the pilot study of Porcaro and colleagues, 30 women with symptomatic UFs were divided into two groups (treated and control). In the treated group, 15 women were supplemented with vitamin D in combination with epigallocatechin-3 gallate (EGCG) and vitamin B6 for 4 months and compared with 15 untreated patients. The results showed that UFs volume decreased by 34.7% in the treated group and increased by 6.9% in control. In addition, the QoL of these patients (31%) significantly improved. The authors concluded that such a combination might be a new form of non-hormonal treatment for women with UFs [75].

## 6. Conclusions

The reported studies describe the effect of vitamin D in counteracting UFs growth, reducing its size, and improving the related symptomatology. Although the mechanisms by which vitamin D exerts its effects in UFs are conveyed through the regulation of gene expression, some of these effects are also mediated by the modulation of intracellular signaling pathways, thus suggesting that vitamin D is directly or indirectly connected to multiple cellular processes. Some examples are reported in Figure 1.

Collectively, some issues emerged from these in vitro studies. Major limits include the concentration of vitamin D, with minimal effects observed at physiologic concentrations; the absence of biomarkers to prove specific on-target response, no studies identified VDR target genes in UFs by ChiP sequencing; the paucity of data regarding the molecular effects of vitamin D, comprehensive system biology approaches to build integrated networks are still missing.

The complexity of UFs in terms of specific cancer subtypes should also be considered. Many results obtained in vitro or using preclinical models may fail to translate into clinical practice where the specific genomic profile of UFs or the presence of multiple extracellular stimuli may influence vitamin D signaling. Moreover, identified abnormalities in vitamin D metabolism, i.e., altered activity or SNP of metabolic enzymes, raise the question as to whether these enzymes contribute to the regulation of vitamin D signaling in vivo. Overall, this suggests a genotype-based clinical strategy aimed at identifying mutations that may have a significant impact on vitamin D action in UFs.

In conclusion, while preclinical data suggests that vitamin D results in molecular alterations in leiomyoma cells, the data supporting clinical benefit is limited and experimental. The available clinical studies [70,71,72] are small (<50 subjects per group). Ciavattini [70] is not randomized, using subjects that refused therapy or “did not perform the therapeutic intervention properly”. Vitamin D supplementation does increase vitamin D levels to marginally normal concentrations (36.1 ng/mL [71] and 30.6 ng/mL [72]) in the two randomized studies. In one, normalizing vitamin D decreased leiomyoma size by 7 mm, while the control group had no growth [71]. In the other study, normalization of vitamin D levels had no impact on leiomyoma size, but the control group had increased growth [72]. For women suffering from symptomatic leiomyomas, the current clinical data are insufficient to support the use of vitamin D at an efficacious therapy. An ongoing RCT may eventually shed light on the role of vitamin D on UFs growth in reproductive stage women by addressing some limitations of the present studies [76].
